# Critical theory and the scholarship of medical education

**DOI:** 10.5116/ijme.5780.e0e5

**Published:** 2016-07-29

**Authors:** John Edward Sandars

**Affiliations:** 1 Academic Unit of Medical Education, The Medical School, The University of Sheffield, UK

**Keywords:** Critical theory, scholarship, medical education

## Introduction

The essential aspects of the scholarship of medical education are reflection and research of the endeavour of medical education.^1^^,^^2^  The endeavour of medical education covers a range of activities, from making policy decisions to curriculum design and  its implementation.  All of these activities should prompt the medical educator to reflect on the purpose of education and the methods that are used to achieve this purpose.  Questions about the purpose and methods are likely to include: Why am I providing medical education in the way that I do?  What are the influences on the medical education that I provide? Are there alternative ways for me to provide medical education?  The answers to these questions will quickly highlight the influence of different beliefs and assumptions held by a variety of powerful stakeholders, including governments, professional organisations and higher education institutions, on the activities of all medical educators. 

In this article, I will discuss the importance of critical theory to inform the scholarship of medical education and how a critical theory perspective can lead to transformative change in the endeavour of all medical education.

### Critical theory and education

Critical theory is concerned with the distribution of power in social systems and how one group in society can be controlled and oppressed by another group in society.^3^  Karl Marx is often credited with the origins of critical theory in the mid nineteenth century and one of his major concerns was how a rich industrial capitalist elite exerted power over the education of workers and their children.^4^ This theme of socio-economic and political control through  education, especially its influence on the development of literacy skills, was  continued in the mid twentieth century by the work of Paulo Freire in Brazil.^5^  

The focus of critical theory and education in the late twentieth and early twenty-first centuries has now widened to include the social justice aspect of marginalised groups in education, with oppression related to socio-economic status, cultural background, gender and sexuality.^4^   This oppression is usually not overt but occurs through the social process of hegemony, in which the influence of the powerful elite is slowly accepted and taken for granted by society.^6^ 

### The importance of critical theory in medical education

A scoping literature search reveals scant reference to critical theory in medical education but there are numerous current concerns in medical education that have a critical theory focus. I present two illustrative examples to show how critical theory can inform an understanding of medical education:

#### a.     Competence based curricula and assessment 

The growth of competence based assessment has a clear reductionist view of clinical performance and there have been numerous curriculum statements that support the narrow development of competences that need to be developed and assessed.^7^^,^^8^  However, clinical performance is holistic and requires the skilful integration of several competences.^9^  Important questions to consider are: Why has there been an almost relentless growth in competence based approaches to medical education, with repeated assessments at both undergraduate and postgraduate levels, but with less emphasis on holistic approaches? What are the consequences for learners and teaching that is directed to a narrow band of competences?  Who are the dominant forces that have influenced this competence based approach?

#### b.     Differential achievement of students 

There are concerns about the differential achievement of black and minority ethnic students (BME) at medical school.^10^ A recent survey at three UK medical schools identified that more BME medical students had a lowered feeling of belongingness compared with their Caucasian counterparts.^11^   Belongingness is an important predictor of  retention of students and academic performance.^12^   Important questions to consider are: What are the factors in the social environment of the medical school that influence the belongingness of BME students?  How do these influences occur in the formal curriculum, the hidden curriculum and in the daily interactions between BME students and their peers and teachers?

Readers of this article are invited to consider questions that arise from their own experiences, with the intention of raising awareness of the influence of power elites in their own context. Without this awareness raising step the current situation is taken for granted and will continue to be replicated.

### Using a critical theory perspective for transformation in medical education

Awareness of the influence of power is only the first step of using a critical theory perspective in the scholarship of medical education.  A process of transformation of the current situation has to occur, otherwise there will be replication of the oppression by dominant elites.  This transformation process requires enquiry based methods to develop greater understanding of the influences within a particular context and empowerment of individuals to make the necessary changes.  These methods include critical policy research, practitioner action research and critical reflective practice, but these methods appear to be rarely used in medical education research.

Critical policy research has a clear focus on understanding how education policy is created, implemented and evaluated.^13^ The research is mainly qualitative and seeks to identify the variety of beliefs and assumptions of policy makers and how they are translated into policy, the persuasive approaches used to implement policy, and the impact of the policy on the range of stakeholders. The findings can be used to raise public and political awareness of the key issues, as well as to inform change in future policy.

Practitioner action research has a more local context focus and seeks to understand and change a social system by repeated cycles of identification of issues, implementation of small changes to address the identified issues and evaluation of the impact of the changes on the issues.^14^

Critical reflective practice is informed by the notions of single loop, double loop and triple loop learning.   Single loop and double loop learning are the usual approaches to reflective practice. Single loop learning is concerned with making reactive changes to practice, in a similar approach to altering the thermostat to the temperature of the room.^15^ Double loop learning has a focus on understand the situation in greater detail, such as why the temperature was not adjusted by another person or why the room temperature has to be maintained at a particular level.^15^  However, the frequently ignored  critical aspect of  reflective practice is triple loop learning, which is concerned with the underlying issues of power that influence the actions within a particular context.^16^ For example, there are important considerations in the thermostat example about the powerful influences that determine the barriers for taking responsibility for decision-making and taking actions.

## Conclusions

Critical theory is an essential perspective to inform the scholarship of all medical educators, although it appears that there is little literature on its application to medical education.  We recognise that healthcare is highly influenced by the socio-political and economic environment. However, we do not appear to have a similar recognition that decisions on policy and practice for medical education are also influenced by the same factors. I hope that this article has stimulated medical educators to begin the essential process of applying a critical theory perspective to their scholarship of medical education, from undergraduate medical students to training of junior doctors to continuing medical education. I believe that it is only by medical educators integrating a critical theory perspective within their overall educational scholarship that they will achieve their professional potential as educators.

### Conflicts of Interest

The author declares that they have no conflict of interest.
